# The Influence of Psychosocial Factors on the Successful Formal Education Trajectories of Young Spanish People in Vulnerable Situations

**DOI:** 10.3390/bs14040342

**Published:** 2024-04-18

**Authors:** Edgar C. Campos, Susana Fonseca, Deibe Fernández-Simo, Carlos Rosón

**Affiliations:** 1 School of Education, Polytechnic University of Viseu, 3500-155 Viseu, Portugal; susanafonseca@esev.ipv.pt; 2 Area of Theory and History of Education, University of Vigo, 32004 Ourense, Spain; jesfernandez@uvigo.es; 3 Igaxes, 15707 Santiago de Compostela, Spain; carlos.roson@igaxes.org

**Keywords:** youth, vulnerability, psychological and social well-being, academic achievement

## Abstract

Young people in vulnerable situations tend to have more difficulties realizing successful formal education trajectories. It is extremely important to conduct studies that explore the key dimensions that can help young people overcome the conditioning of vulnerable situations, assisting in the achievement of academic success. According to the existing literature, one of these key dimensions is psychosocial well-being. Thus, this qualitative research aims to identify and analyze psychosocial factors that favor successful school pathways among young people in vulnerable situations. Through a case study approach, 12 in-depth interviews were carried out with 12 young Spanish people who not only have a vulnerable background but also successful formal education trajectories. The data were explored through qualitative content analysis—NVivo11. The results revealed that young people generally associate their successful educational trajectory with the positive impact of psychosocial factors, such as the following: positive caregivers’ valuation of formal education and positive school experiences and support networks. Young people also point to many negative school experiences that have been overcome via positive attitudes and individual protective factors. The youngsters suggest changes in the educational system and teachers’ skills in order to improve the levels of psychosocial support in formal education contexts. The study’s implications and limitations and recommendations for future research are discussed.

## 1. Introduction

Formal education is a process that presupposes teaching and learning and serves to assess and certify individuals’ scientific and academic competencies [1]. It encompasses all education typically provided in schools, educational centers, and training centers [2]. According to *Ministerio de Educación*, *Formación Profesional y Deportes* (MEFPD) of the *Gobierno de España*, the Spanish educational system is divided into the following: *Educación Infantil* (0–6 years); *Educación* Primaria (6–12 years); *Educación Secundaria* (*Educación Secundaria Obligatoria* [ESO]—12–16 years; *Bachillerato* or *Grado Medio*—about two years after ESO); *Educación Superior* (*Enseñanzas Artísticas Superiores*, *Enseñanza Universitaria* [*Estudios de Grado*, *Máster y Doctorado*]) [3]. Alongside these educational levels, it is possible to pursue vocational (*Formación Profesional* [FP]), artistic, or sports training studies that confer technical diplomas at the intermediate and higher levels (*Grado Medio* and *Grado Superior*) [3]. Vocational training [FP] can also be undertaken during the period of Compulsory Secondary Education (ESO) [3]. Formal education failure and success are phenomena with very different meanings depending on the social, economic, political, and individual contexts in which they occur. Specifically, in the Spanish context, some authors associate formal education failure with the non-completion of Compulsory Secondary Education or school dropout [4,5]. However, other authors state that formal education failure does not only materialize in the retention, non-completion, or abandonment of the school trajectory but also in the scarcity and/or absence of opportunities for developing personal and social skills and successful experiences in the school context [6,7]. Nortje and Pillay, in their study on young people in vulnerable situations, highlight experiences of formal education failure, which are expressed as failures in psychosocial support provided by the school, especially the lack of understanding of their vulnerable situation, lack of a sense of identity, emotional stress, lack of vocational guidance, social isolation, and lack of social skills [8].

At this point, we underline that a situation of vulnerability—with essentially personal and social meaning—refers to situations of risk and danger related to particularly challenging personal characteristics and/or the lack of family, community, and/or social support. All this can translate into the fragility of affective and identity ties, discrimination, and/or difficulties in accessing social, economic, and cultural opportunities [9,10,11]. 

Montserrat et al. stated that what happens at the school level or involves the school environment has a very relevant weight in the lives of particularly vulnerable young people [12]. Young people from more vulnerable socioeconomic, family, and relational contexts are closer to failing formal education trajectories [13,14,15]. In fact, situations of vulnerability are a strong impediment to achieving successful experiences in the school context [16,17]. Therefore, the consequences of formal education failure, with a particular focus on achieving an autonomous and fulfilled life, have a decisive weight on young people’s futures, particularly those who are in vulnerable situations [9,18,19].

On the other hand, formal education success is related not only to obtaining good grades but also to knowledge, skills, and attitudes that prepare students for the challenges of society and life, recognizing their uniqueness and autonomy, in addition to responsibility, proactivity, critical sense, and happiness [7,20,21]. Salva-Mut et al. consider that formal education success depends on factors that cut across the individuality, relationships, and educational institution of each youth, such as the comprehensive and personalized approach taken, the contents learned, the methodologies used, the emotional bond, the distinct environmental aspects of the educational context, and the positive impact of participative experiences at the personal and work levels [22]. Kelly et al. point out that positive formal education experiences, specifically at the social and emotional support levels, are crucial to achieving positive experiences after the school itinerary, contributing to emotional and economic well-being [23]. Essential factors such as health, housing, economy, family, and formal, non-formal, and informal social support networks contribute to youth’s formal education success, especially youth in vulnerable situations [20,24]. Complementarily but more comprehensively, Garcia-Molsosa et al. associate the formal education success of students in vulnerable situations with factors such as individual characteristics, students’ academic aspirations and strategies, relationships with classmates and teachers, educational experiences, the complexity of structuring, the students’ sense of belonging to formal and informal support networks, and political actions and investment in inclusive education [25].

In this context, it is important to talk about psychosocial factors, which means a set of elements that influence psychological and social well-being that in turn have an impact on individuals’ development, perceptions, behaviors, attitudes, beliefs, and interactions with society [26]. Some of these factors can include socioeconomic and cultural background, personal resilience, family dynamics, peer relationships, and formal and non-formal support networks [27]. Regarding the context of formal education, Fennie et al. consider psychosocial factors, such as transition support, social interaction, help-seeking capabilities, and motivation levels, as important to academic achievement [28]. The conclusions of Mihaela’s study indicate that academic success is not only related to intellectual skills but also to personality traits, such as emotional sensitivity, expansive tendency, and assertiveness [29]. According to Ratanasiripong et al., academic success depends on family relationships, emotional negativity, and self-esteem [30]. Tindle et al. found correlations between academic performance and “social support, motivation, stress, satisfaction, self-efficacy, anxiety and depression, prior academic achievement, study effort, adjustment, family, and emotions” (p. 1) [31]. With a sample of students in situations of social vulnerability, Swanson et al. concluded that academic success is related to psychosocial factors such as a sense of belonging and self-efficacy at academic and social levels [32]. 

According to Krovetz, resilience is a key supportive factor for children and young people in vulnerable situations [33]. Bernard says that social competence, problem-solving skills, autonomy, and a sense of purpose and future are characteristics that are closely related to resilience [34]. Worrell says that resilience makes students in vulnerable situations and at risk of academic failure more predisposed to being talented students [35]. A study by Martin et al. asserts that resilience mitigates the effects of negative educational outcomes [36]. Ungar et al. state that the school, along with the family and the community, plays a fundamental role in empowering resilience in children and young people in situations of vulnerability [37].

In 2022, the Directorate-General for Education, Youth, Sport and Culture of the European Commission published a “Proposal for a Council Recommendation on Pathways to School Success”, in which the following objectives are highlighted: educational success cannot depend on socioeconomic and cultural levels; we need to reduce the proportion of students with low achievement and those who drop out early from education and training; we must promote inclusive education and training, with values such as equity, quality, academic performance, involvement, well-being at school, mental and physical health, and respect for diversity; we must develop, through mutual learning, a shared understanding of the factors that promote educational outcomes and well-being, with particular focus on students from disadvantaged backgrounds [38].

The present study is precisely focused on this last aspect (focusing on the formal education itinerary of students in vulnerable situations), and it focuses on the few studies that have attempted to identify and analyze young people who, despite their vulnerable situation, have managed to achieve academic success [4,5,7,10,12,14,15,20,22,24,39,40,41,42]. Notwithstanding the existing work in this area, several studies point to the need to continue conducting research that explores the dimension of students in vulnerable situations and the factors associated with their academic performance [24,43,44,45]. Faldet and Nes emphasize the need to continue conducting research in education, giving voice to students in vulnerable situations [46]. Conducting studies on this subject and this specific population is relevant due to the markedly subjective and continuously changing nature of this reality. Thus, the aim is to individuate and analyze the psychosocial factors that are part of this formal education success itinerary via the qualitative content analysis of young people’s perceptions.

## 2. Materials and Methods

### 2.1. Study Design 

#### 2.1.1. Qualitative Approach and Research Paradigm

We designed a qualitative study to analyze the successful formal education paths of young people in vulnerable situations, identifying and exploring the psychosocial contributing factors. In this sense, we decided that listening to the voices of young people who share positive and negative aspects of their formal education would favor a deeper understanding of their psychosocial experience [47,48,49,50,51]. The collected data seek to promote reflections on the personal and social characteristics, approaches, and actions that contribute the most to vulnerable young people’s formal education success. The proposed research study attempted to promote not only young people’s self-assessment of their academic results but also the contemplation of all relationships and educational structures and supports that accompanied them on their formal education trajectories and that are an integral part of their psychosocial world [52,53]. We chose a case study approach because young people, even though they are from different regions of Spain, belong to the same federation of entities [54]. Other studies have examined the perspectives of young people in situations of social vulnerability regarding their academic pathway using qualitative research, notably the case study approach [4,5,45,55,56]. Thus, our study fits within the constructivist paradigm because we aim to explore and interpret the meanings that participants construct about themselves, their experiences, and their social interactions [57]. Therefore, we seek to obtain a comprehensive understanding of the phenomenon under study from the data emerging in the research process [58]. 

#### 2.1.2. Researchers Characteristics and Reflexivity

As described in this section, data collection was carried out by professionals who were directly connected to the young people, increasing the availability of participation and the depth of contributions. All authors have had direct contact with young people in situations of social vulnerability and with institutions that have cared for children and young people throughout their professional careers. This fact suggests that the analyses and interpretations of the results may be more reliable [59].

#### 2.1.3. Context and Sampling Strategy

The selection of participants was limited to the specific context of the *Jóvenes e Inclusión Social* Entities Federation. The tendency to achieve academic success is lower among young people in situations of social vulnerability [16,17]. It is very challenging to secure the participation of that young people. Having the participation of some of them is very enriching for the understanding of this topic. Therefore, a convenience sampling strategy was used [60]. General inclusion criteria were defined as follows: living in Spanish territory; being or having been in a situation of social vulnerability (young migrants, young people with neurodevelopmental disorders, and/or those who have received care from child protection entities); having or having had a connection to an institution linked to minors’ protection and/or migrants’ reception; having or having had a trajectory of academic success. 

### 2.2. Ethical Issues

The ethical principles for this type of research study were safeguarded and respected at all stages of study development, with integrity conferred by the Ethics Committee of the Doctoral Program in the Education and Behavioral Sciences of the University of Vigo, and the approval code is CE-DCEC-UVIGO2023-06-14-0872. All participants were provided with prior knowledge of assumptions, objectives, and study implementation methods. All participants and/or legal guardians signed the respective informed consent before commencing data collection. The anonymity and confidentiality of data were always ensured. Participants were reassured of the option to withdraw from the study at any point in the research process. Participants were assured of the opportunity to access the study results at the end of the data collection and analysis process. Data collection occurred in environments that ensured maximum privacy.

### 2.3. Data Collection Methods, Instruments, and Technologies

We collected qualitative data from the youths’ sharing of their perceptions through individual semi-structured interviews. Data collection was conducted between 2022 and 2023. A semi-structured interview guide was constructed ad hoc [49,61,62,63], with questions based on some of the existing works that relate school success to supporting psychosocial factors [1,2,13,18,20,27,28,29,30,31,32]. The questions addressed the transversal dimensions of the participants’ characteristics and experiences, such as the following: (1) sociodemographic dimension; (2) family and growth context; (3) formal education context; (4) school experiences; (5) relationships and formal, non-formal, and informal support networks; (6) evaluation of the Spanish Educational System; (7) personal characteristics contributing to their formal education success; (8) plans for the future. The semi-structured interview guide allows both the participant and the interviewer to adjust the composition and organization of the questions, also allowing for the exploration of new topics [64]. However, our initial guide had the following questions: Who are you? How old are you? What are your origins? What is your family like? Where do you live and what is your current occupation? Did your parents or caregivers give importance to your studies? What about your school? Do you feel good at your school? Who are the most important people to you in the school context? Do you have a teacher, school professional, or classmate who is a reference for you, or a professional from the social support institution? Why are these people important to you? What positive and negative experiences have you had in school? What are the biggest obstacles or difficulties you face in school? How did you overcome these difficulties and obstacles? What do you think about the Spanish Educational System? Would you change anything? What are the characteristics that have helped you achieve academic success? What are your plans for the future?

All participants were previously informed about the purpose of the research study and the conditions for conducting the interview (using a voice recorder) [49,61,63]. Likewise, all participants or legal guardians signed an informed consent form [63,65]. The interviews were carried out individually in comfortable locations by specialized professionals linked to the participants, attending to confidentiality and privacy [62]. All professionals who performed these interviews knew the respective participants, facilitating the researcher’s acceptance and allowing for greater depth in the answers [59]. 

### 2.4. Participants

Twelve young people participated, comprising six females and six males linked to the *Jóvenes e Inclusión Social* Entity Federation. Table 1 presents the sociodemographic data of the participants. The average age was 21.5 years. Only one participant was a minor, for whom authorization from the legal guardian was requested. Most participants were born outside of Spain. All participants attended some training or educational path. Those exclusively working had recently completed their training. Three participants are attending or have completed university degrees. Even though many participants have already started working, they continue with their educational pathways.

### 2.5. Data Processing and Analysis

The total recording time was 7 h and 38 min, with an average time per interview of 38.17 min. The recordings have been transcribed. The data were analyzed with the qualitative content analysis technique using NVivo 11 software. This technique allows for the quick and comprehensive analysis of large volumes of data; it is a systematic analysis procedure that is guided by rules and quality criteria, allowing for evidence corroboration. It is a process characterized by clarity, objectivity, and rigor in analysis, and it is a flexible technique that allows for adjustments and reformulations. Moreover, it promotes the deepening and attribution of meaning to the entire analysis, as it leads to the production of inferences about the content [49,66]. For the qualitative content analysis process, we used the structure proposed by Bardin with the following phases: (1) pre-analysis (elaboration of objectives and indicators and material preparation for analysis); (2) material/data exploration (readings, clippings, classification and aggregation, anonymization of the excerpts, and coding/categorization); (3) treatment/analysis of results (inferences and interpretations) [67]. In the codification process, codes and subcodes were created according to the inductive method [66,67,68,69,70,71]. The qualitative content analysis technique has some risks: dependence on subjective interpretation; words and codes being polysemous and ambiguous; overlooking less frequent codes and valuing only frequent codes; dependence on the representativeness of the content of the data used [49,50]. To overcome these risks, the first author coded the data and, after this phase, discussed the codes and subcodes with the other authors, reaching the final version [66]. The codes and subcodes created were also confronted with the supporting bibliography and previous results [72]. The sample size does not allow for the generalization of results, which can be carried out in quantitative research, but it likely allows for generalization through transferability as described by Smith. Indeed, the case study approach leads us to consider whether the data are applicable to the specific context of the analyzed federation of entities and whether the results can be extended to other similar realities [54,73]. 

## 3. Results

The following table (Table 2) shows the number of references by codes and subcodes according to the qualitative content analysis of young people’s responses. For each code and subcode, coverage is assumed (number of respondents who contributed to it). The codes and subcodes refer to the psychosocial factors that, according to the respondents, influence or have influenced their formal education trajectories.

The table’s results show that positive experiences in school, particularly successful experiences in studies and positive relationships with teachers or school professionals, are the most significant psychosocial factors determining academic achievement. Family, friends, care institutions, and the professionals within these institutions form a fundamental social support network that, together with caregivers’ positive reinforcement of education, is also crucial for academic achievement. There are many reports of negative experiences in school, both in established relationships and in failures in studies. Various obstacles with the potential to hinder academic achievement have also been reported. However, there was less coverage from respondents in these areas. The more negative aspects that young people faced did not outweigh the positive school experiences and social support networks. Here, the personal characteristics that helped overcome negative experiences seem to have gained importance. Some young people think that significant changes should be introduced in the educational system and in the skills and approaches of teachers to create school environments with improved psychosocial support. Although plans for the future of young people largely involve uncertainties, the academic success they have experienced leads them to consider work, further education, and relationships as goals for their vital itinerary.

### 3.1. Caregivers and Respondents’ Valuation of Formal Education

The answers imply that a successful formal education itinerary largely depends on the caregivers’ understanding and supportive attitude regarding the importance of formal education, and the effects of their support span multiple levels. In this regard, youngsters stated the following: “*They taught me a lot about studies when I was little*” (E4); “*They always considered it very important. That is*, *we should go to school every day, be punctual, study, do our homework* (…) *They emphasized studying to be good professionals in the future* (…) *They always supported me in my decisions*” (E9); “*They always supported me a lot, provided me with comprehensive support for many things, either concerning my studies or personality*” (E10). We can summarize this supportive and understanding attitude of caregivers as follows: awareness of the importance of education; constant encouragement in achieving academic milestones; closeness in times of failure and recognition of successes; and support, involvement, and joint reflection on decisions regarding the academic path. On the other hand, there are caregivers who do not provide ongoing support, which can make young people feel that formal education is not important: For example, “*Yes*, *my father was concerned about my studies. But then, over time*, *he gradually lost interest, and in the end, he did not even buy the study materials and that was what led me to decide to drop out of my studies*” (E6) is one of the most significant contributions relative to subcode “low support and valuation”. Although there are answers that refer to the appreciation of young people regarding the importance of formal education in their own lives, as we see in statements such as “*Because without education you won*’*t get the job you want*” (E7), the answers focus much more on the influence of caregivers who value or do not value formal education. 

### 3.2. Positive School Experiences

Ten young people reported positive school experiences, especially concerning good relationships established with teachers or school professionals (“*During the first three months of the course, they are very concerned about whether you integrate in the school. You can go in the afternoons to talk to the teachers*, *have separate tutorials; there are many coordinators who worry if something happens to you; that is, the relationships much closer*” [E2]; “*At the University, there was a professor who understood that it was difficult to combine work with studies*, *so*, *if you explained it to him and said ‘Look, I can’t do this tomorrow because I have to work’… he understood* [E5]) and experiences of success in their studies (“*It was satisfying in the end because you recognize the effort you have made and your ability*, *what a good student you are*” [E8]; *In high school, I already improved because I began to understand more what it is to study* (…) *I organized myself well and improved my grades a lot* [E11]), linking them to their successful formal education trajectory. The feeling of academic competence and the establishment of relationships with teachers who have understanding and open attitudes towards vulnerable situations seem to be a determining psychosocial factor in reinforcing young people’s motivation and interest in their educational trajectory. The positive attitudes of teachers and other school professionals that support the academic path of young people can be summarized in the following actions: understanding vulnerability; availability for guidance and accompaniment; sensitivity; and flexibility. The experiences of success in studies appear to have provided young people with psychosocial reinforcement through higher levels of self-efficacy and personal satisfaction, which have become motivations to pursue further studies with dedication. Good relationships with classmates are also considered an important psychosocial factor, albeit with less relevance.

### 3.3. Negative School Experiences

When talking about formal education success, we could be led to think that young people’s negative experiences in school are rare. However, respondents shared several situations that on a psychological and social level made the vulnerable situation more difficult to bear. For example, the subcode “about failures in studies” is well represented by the expression “*I have rather bitter memories of sitting every afternoon with my mother trying to learn the subject and I could not*” (E3). It is also very worrying that young people in vulnerable situations have experienced discrimination, judgment, exclusion, and even bullying perpetrated by their peers, as the following statements report: “*The children, at least as I saw it, laughed at me because I did not know how to talk*, *because I am different, because children attack you through these things*” (E4); “*It was a little difficult for me to go to class, because I was, well, like the* ‘*daughter of…*’”(E5). Serious situations were reported in which some teachers or school professionals demonstrated difficulties in dealing with the vulnerable situation of young people, and this had heavy repercussions in areas such as self-esteem and self-confidence: “*This made me not want to continue studying; they humiliated me in front of the class for writing very badly or for not having learned my lesson*” (E3); “*And the teacher was angry*, *she wrote notes to my parents*, *and she couldn*’*t think of anything better than to dictate it out loud, in front of all the children. I felt very ashamed*” (E4); “*I felt quite criticized and judged by the teachers and with no support, I heard expressions like* ‘*the kids from the center*…’” (E10). In the “negative school experiences” code, there is a very similar distribution of frequencies in various subcodes, creating situations of failure in relationships that are particularly felt by young people in vulnerable situations. Even though the number of reported negative experiences is very high and this psychosocial risk factor is harmful, especially for these young people, the number of reports of positive experiences is clearly higher.

### 3.4. Personal Characteristics That Lead to Success

The personal and social skill dimension inherent to each young person is one of the most considered psychosocial factors in realizing successful formal education trajectories. This means that there are characteristics, which we can call individual protective factors, and positive attitudes in facing difficulties, which can be materialized in quoted behaviors such as the following: “*Sometimes*, *you have to dare and*, *if you make a mistake*, *you will correct it*” (E1); “*Flexibility, that is, I adapt very quickly to things*” (E2); “*I also did it with all my effort and with my desire*” (E6); “*I was like: ‘You*’*ve come this far, you*’*re not going to drop it in the middle. You have to finish!*’” (E8); “I *had to adapt to the personalities and customs of other people who had nothing to do with me*” (E10) “*I grant a lot of importance to my mentality and organization*, *my autonomy, my desire*” (E11). We could summarize these characteristics in expressions as follows: autonomy, self-confidence, resilience, motivation, willpower, persistence, responsibility, flexibility, and adaptability.

### 3.5. Support Networks

According to most respondents, achieving formal education success in a vulnerable situation is not something that can be carried out alone. The family’s support, even when it is not regular, and the support given by friends and institutions constitute a network that provides security in moments during which obstacles need to be overcome. This trend of responses may be related to what young people have already emphasized about the importance of caregivers’ value of formal education. Regarding family and friend support, some young people stated the following: “*Factors external to me*, *it may be my family*, *who has always been there supporting me*” (E9); “*The environment is very important; that is*, *not only those who support you*, *like a family, parents and so on*, *but also for me, a very important factor are my friends*” (E10); “*I can include my relatives because they have supported me and always respected my study goals*” (E11). Regarding institutions and professional support, some respondents said the following: “*That*’*s when I went to family therapy with my family*” (E3); “*Very good*, *because of the educators who were there*” (E5); “*In the center, I saw myself as really wanting to study, to do things well*” (E6) “*What they are doing for the neighborhood is very good*, *mainly for all the families* (…) *If I weren’t for that entity*, *I might not be here*” (E9). We can summarize these psychosocial factors as the perception of the presence of family and friends with attitudes of respect, companionship, and support relative to school and learning. Similarly, institutions that truly embrace the characteristics of young people and their families, proposing effective intervention strategies to minimize the impacts of vulnerability, are observed as enhancers of educational pathways. The trust and close relationships between professionals and young people are also considered fundamental to academic achievement.

### 3.6. Formal Education Obstacles

The young people highlighted some obstacles to their educational itinerary, denoting, in various reports, a certain sense of social exclusion and misunderstanding of their vulnerable situation. Young people who were born outside of Spain indicate difficulties, for example, with legal issues, language, or simply because they are from a foreign country, which creates situations of psychosocial instability. For example, some report the following: “*I also had a lot of difficulties with the papers*” (E7) or “*I kind of had a lot of things going against me to begin with: being a foreign woman, from flats. In the end, it was like I had to overexert myself so as not to be at the same level as others*” (E10). Being someone who has a vulnerable background is felt by young people to be one of the biggest obstacles, as witnessed by a young girl who reported the following: “*My family history was something that made it difficult for me throughout my studies*” (E5).

### 3.7. Suggestions to Favor Psychosocial Support in Formal Education Contexts

In the following two reports, young people suggest changes in the structuring of the educational system, focusing attention on the specificities of each student, providing more support to those who are in vulnerable situations, and removing the pressure of having to make decisions too quickly: “*Special attention to each kid is very important. The classrooms are very large. You can’t ask a teacher to consider the circumstances of each of the thirty kids he has in class, because he has others throughout the course*” (E3); “*It*’*s like “choose and go” (…). There should be more economic resources to support university studies because*, *in addition to that*, *add the scarce family and social support. I mean that there are many difficulties in the end to achieve a university degree*” (E5). In the same way, teachers must strive to understand the specificities of each student, improving their skills in establishing more appropriate relationships and realizing pedagogical actions, as is clearly stated by this young man: “*It should not be the kid*’*s mother or father who has to go and explain to the teacher how he should do things* (…) *It is true that he is the teacher of a class in a standardized Education System, but there will always be dyslexic kids, with attention deficit or other difficulties, and it is very important for the teacher to understand this*” (E3)”.

### 3.8. Plans for Future

There are many young people without a clear idea of the future, as observed in expressions such as the following: “I don’t know. That is, it’s like I find it hard to imagine it, just in case it is not fulfilled” (E9); “I have an idea about the things I would like to do in my professional career and such, but I cannot tell you their order and how it will be” (E10). However, despite not having a clear life plan, young people achieved successful paths in formal education. This fact makes us return to the frequency by which some young people point to support and good relationships in formal, non-formal, and informal spheres as psychosocial factors determining their permanence and commitment to school.

## 4. Discussion

In this study each young person’s context is valued, with their idiosyncrasies, considering the entirety of their responses as a global contribution to identifying and analyzing psychosocial factors that may lead to trajectories of educational success in situations of vulnerability. As shown in the young people’s responses and the codes and subcodes that emerged in the qualitative content analysis process, life trajectories—particularly formal education success trajectories—are not only related to positive aspects of the school environment [25]. These trajectories are also complex and transversal relative to the young people’s relationships and contexts, and they include negative experiences and the mechanisms that overcome them. This aligns with most authors’ studies on the same topic [4,5,15,20,22,41,42].

For the respondents, having had positive school experiences is crucial to achieving formal education success. These experiences include times during which they felt successful in their studies. Prestes et al. refer to the scarcity of failure experiences in the reports of young people who have successful formal education trajectories [41]. In this research, references to successful experiences in studies also prevail. According to our results, the experience of success in studies seems to lead to higher levels of self-efficacy and personal satisfaction, which are motivations for the pursuit of studies with real commitment. The results of Pedditzi’s study highlighted “the presence of low levels of satisfaction with quality of life and self-efficacy in students at risk of dropping out of school” (p. 7) [74]. The same study considered academic performance and satisfaction with school experiences as highly associated with the possibility of dropping out of school [74]. Failure experiences, although they exist, seem to have been transitory and relatively insignificant compared to successful experiences. However, positive school experiences are mainly related to young people’s establishment of beneficial and healthy relationships with teachers or other school professionals, providing emotional and cognitive stability to reinforce their positive trajectory. Kelly et al. reported that the social and emotional support received at school leads to greater well-being [23]. In this sense, the school and its professionals should ensure the students’ psychosocial well-being [8], considering aspects such as their personal and social characteristics and the sociocultural and economic context [21,38,42]. The results of the study by Bruin et al. indicated that “building social capital for youth at risk through developing relationships that generate motivation, trust, and confidence enhances students’ opportunities for participation and subsequent learning” [75]. Although many positive school experiences are listed, the many mentions of negative experiences and obstacles should not be ignored. These negative experiences can lead to great misunderstanding; insensitivity; and in some cases, segregation, social exclusion, and violence, which could be carried out by teachers and other school professionals, classmates, or even encouraged by the functioning of the schools or the educational system. The harmful nature and impact of these actions and dynamics in vulnerable young people’s lives are reported in the studies by Espinoza et al. and Medina et al. [40,76]. It should not be forgotten that young people point to positive relationships with teachers or school professionals as the main positive experience in the school context, with considerably fewer references to relationships with classmates. This clearly shows the teacher and school professional’s importance, increasing their responsibility. From the results, it is evident that actions such as understanding vulnerability, availability for guidance and accompaniment, sensitivity, and flexibility are highly valued by young people. Jopling and Zimmermann refer to this as a collaborative relationship between teachers, school professionals, and young people [43]. The study by Hendrickx et al. demonstrated that teachers who had fewer negative attitudes towards students in situations of social vulnerability led those students to feel more socially integrated in their classmate group [77]. Farmer et al. concluded that students in vulnerable situations need classroom contexts that are non-hierarchical and that provide opportunities for social visibility and the assumption of different roles while ensuring individualized support and the development of new social skills [78]. 

One of the most significant observations from this research study is the positive attitudes when facing difficulties and individual protective factors reported by young people as fundamental characteristics that led to success. We could summarize these characteristics in expressions as follows: autonomy, self-confidence, resilience, motivation, willpower, persistence, responsibility, flexibility, and adaptability. Likewise, Solera et al. and Crous et al. indicate resilience as a predominant factor for achieving formal education success in vulnerable situations [15,18]. Núñez et al. associate young people’s formal education success with self-confidence, willpower, and their ability to take advantage of development opportunities [14]. In the study by Mayayo et al., a responsible personality is essential to compensate for the gaps resulting from the situation of vulnerability [4]. Martins et al. and Salva-Mut et al. underline the idea of autonomy as one of the best predictors of formal education success [21,22]. In this sense, we can say that these psychosocial protective factors have a double function: to cushion the impact of the vulnerable situation and to overcome the challenges encountered throughout the formal education trajectory and in the social panorama.

Another relevant topic mentioned by young people comprises informal and institutional support networks, specifically support relationships with family and friends and connections to institutions and/or professionals. Marion et al.’s study also indicated the importance of social support networks [79]. Caregivers’ good valuation of formal education is a key aspect identified by young respondents as favoring formal education success (more than the valuation made by the young people themselves). In our study, caregivers who are capable of fostering awareness relative to the importance of education; providing constant encouragement to achieve academic milestones; offering closeness in times of failure and success; providing support and involvement; and engaging in joint reflection on decisions regarding the academic path appear to be a significant psychosocial factor in the school journey. The results of Cheung et al. suggested that caregivers who provided more academic support at home and a more positive literacy environment were more likely to care for youth with higher levels of academic success [80]. However, caregiver involvement in school was not significantly associated with the academic performance of youth in vulnerable situations [80]. Crump and Slee already underlined the importance of the family or, eventually, of institutions for vulnerable young people’s formal education success [20]. Thus, there seems to be no formal education success without young people’s strong perception of this support. This is consistent with the statements reported by Schmid and Garrels, indicating that vulnerable young people require informal support that values their school careers, encourages them, and motivates them toward educational success [81]. The study by Gadea et al. reinforces this idea, emphasizing that family and friend groups mitigate the situation of vulnerability in young people’s formal education [82]. Regarding this reflection, young people emphasized the importance of the support of institutions and professionals who were indispensable throughout their successful formal education. In the studies of Mayayo et al. and Lindahl and Trull-Oliva et al., the importance and decisive contribution of institutions and professionals relative to successful formal education trajectories and psychosocial and economic stability are clear [4,83,84]. In the research conducted by Maclean et al., the relationship between high academic achievement and institutional support and services was highlighted, albeit with inconsistencies across the analyses [85]. Trust and closeness with professionals of institutions are particularly relevant for the young respondents, similar to what Silva et al. observed when they stated that high-quality relationships between young people and professionals lead to higher levels of academic success [86]. The study of González-García et al. identified key factors related to the academic achievement of youth in vulnerable situations: “stability (…) in the school, the stable presence of reference adults who are involved, and have expectations of success, along with the involvement of schools in meeting these children’s needs” (p. 9) [24]. 

Regarding suggestions that favor psychosocial support in formal educational contexts, some respondents requested more financial aid to help young people in vulnerable situations complete their studies successfully and without excessive worries. The study of Prestes et al. corroborates this idea, noting that when economic incentives are not provided, most vulnerable young people drop out of school, especially in the higher education phase [41]. On the other hand, several young people suggested significant changes in the Spanish educational system, namely the need for more individualized attention relative to each student, especially students in vulnerable situations, which is in line with the recommendations of several authors [21,22,38,42]. An important respondent contribution at this level also referred to the need to include subjects or activities in the school curriculum that would prepare young people for an independent life, especially regarding financial literacy, integration in the labor market, and social inclusion. González-García et al. consider that these adaptations are extremely necessary, with particular emphasis on non-native youth [24]. The results of the study by Martín-Gutiérrez and Sevillano-Monje point to necessary changes in the Spanish educational system in order to better include vulnerable groups in educational institutions, and these changes are related to educational styles, material and human resources, pedagogical practices, and interactions and relationships with families [87]. No less important were the young people’s comments that referred to the necessary changes needed in teachers’ skills and attitudes, a clear response to negative school experiences with teachers or other school professionals. The study by Christodoulidi discusses the adoption of “pedagogy of vulnerability” as an effective method for teachers and school professionals to create spaces of greater understanding and inclusion for students in these situations [88]. Educational policies should follow these young people’s suggestions and be guided by their sense of empowerment, particularly regarding people in vulnerable situations, to increase individual potential, provide equal access to opportunities, and reduce the risk of social exclusion [89]. Regarding necessary changes in the educational system that foster the educational success of youth in care, the findings of the study by Rutman and Hubberstey “highlighted the importance of a relationship-based approach, including someone who tracks and supports attendance; stability in schools, placements and community; wrap-around model; and extracurricular program” (p. 257) [90]. Themelis and Tuck report that education systems cannot be conceived as challenging, disheartening, and frustrating realities but as sites of promise, opportunities, and possibilities [91].

Another very relevant fact is that when asked about plans for the future, young people substantially tended to express doubts or use inaccurate definitions. According to Romaní et al., there would be fewer uncertainties about the future if these young people were not in situations of vulnerability, which result in prolonged periods of suffering and limit their access to development opportunities [5]. In the same sense, López et al. state that it is more complex to dream of a stable future for young people in vulnerable situations [92]. It is very interesting that, despite having many doubts about the future, participants manage to have successful formal education trajectories. This fact reinforces the idea that psychosocial factors related to personal characteristics and formal, non-formal, and informal support networks are decisive in achieving formal education success, overcoming the obstacles that block the path of educational success and that are inherent in vulnerable situations.

## 5. Conclusions

This qualitative research study, carried out using a case study approach, has helped consolidate important messages and concerns regarding the formal education of youth in vulnerable situations and who belong to a specific federation of entities. Through the sharing of the youth’s experiences, this study unveils novel themes concerning the psychosocial factors that contribute to academic success. The formal education trajectory of young people in vulnerable situations has limitations and constraints that make it difficult to achieve success. However, some young people overcome personal, family, and contextual weaknesses and succeed in school. Young people predominantly associate school achievement with positive school experiences, particularly relationships with teachers or other school professionals, and success in studies. In addition to these data, the formal education success of the interviewed young people also seems to be related to the informal and institutional support networks observed in the positive relationships with family, friends, institutions, and professionals from these institutions. Parents or caregivers who positively value formal education are fundamental to achieving successful trajectories. Many negative school experiences and obstacles are also reported. There are still teachers, classmates, school professionals, and structures that, besides not understanding vulnerable young people’s situation, engage in behaviors of segregation and discrimination. Some positive attitudes to facing difficulties and individual protective factors seem to help young people in vulnerable situations overcome trauma and negative school experiences and focus on their own success story: autonomy, self-confidence, resilience, motivation, willpower, persistence, responsibility, flexibility, and adaptability. The young interviewees suggested significant changes in the Spanish educational system, with the clear objective of increasing psychosocial support in formal education contexts: more economic support for vulnerable young people, more individualized attention relative to young people’s characteristics and contexts, teachers with more skills and more positive attitudes in dealing with vulnerable situations, promoting the “skills for life” acquisition, and the improvement of academic skills. When asked about the future, young people tend to have doubts or vague ideas, a fact that did not prevent them from achieving success due to having good psychosocial support.

### 5.1. Implications

It is essential to create a positive relationship between formal, non-formal, and informal education [52,53]. These integrated social support networks are one of the key characteristics in pathways to academic success among students in vulnerable situations [93]. The results indicated that, at times, school is a space where exclusion occurs, and there is a lack of understanding of students’ situations and a failure to convey knowledge and psychosocial skills that would enable the ability to cope with vulnerability and its effects. This implies that schools and their professionals need to have appropriate mechanisms, tools, and training to deal with situations of vulnerability, which would allow the application of innovative strategies that foster academic success among students [94]. There are examples of evidence-based programs or interventions that help develop skills, both for teachers and professionals as well as for students, which in turn create school environments with greater psychosocial support [95]. The CALMERSS program is effective in increasing well-being and self-care levels and reducing psychological and physical tension among teachers and educators [96]. The ACHIEVER Resilience Curriculum program has demonstrated its effectiveness in reducing occupational stress and increasing teachers’ perceived self-efficacy [97]. RULER is an evidence-based approach that allows for the creation of more positive school climates by valuing emotions and strengthening emotional intelligence in students [98]. Project ACHIEVE is an evidence-based school effectiveness and school improvement program focusing on the academic and social, emotional, and behavioral attainment of all students [99]. Interventions with young people in vulnerable situations should contribute to the creation of contexts and the acquisition and improvement of skills that firmly contribute to young people’s psychosocial well-being, which are fundamental for educational success and healthy development [100,101]. The Spanish educational system must adapt to the demands of all students. Schools could adopt methods to foster a sense of connection among young people in vulnerable situations [102].

### 5.2. Limitations

The results of this research are, above all, applicable to the specific reality of the youth belonging to the Federation of Entities *Jóvenes e Inclusión Social*. Due to its particularistic and subjective nature and approach, generalizing the results to situations beyond those addressed in this study becomes difficult [103]. That was not the purpose of this research study. Nevertheless, the expectation is that the conclusions may be useful in other similar contexts and that similar relationships may be found in other personal and professional experiences, as well as in other studies, which is in line with generalization by transferability as described in Smith’s study [73]. Although the subjective dimension is valued in this research study, samples with a larger number of participants could provide broader coverage and an understanding of the phenomenon under study. In the same way, integrating quantitative methods could enrich the study, allowing not only a deepening of individual experiences but also the identification of general patterns or trends. Acknowledging the subjectivity of participants’ shares and interpretations in studies entails the risk that other individuals experiencing similar situations may not share the same perspectives and may not identify with the results. Discussing results by comparing them with other studies, even if similar, also constitutes a limitation, as the assumptions of the contexts involved may diverge with respect to many characteristics and variables. 

### 5.3. Suggestions for Future Research

Replicating these analyses in as many contexts as possible may lead to an increase in better adaptations in the educational system and its interventions. Therefore, the continuity of this type of study is suggested, which values the subjective dimension and group idiosyncrasies in scientific research processes. Including the perspectives of education professionals, such as teachers, social educators, and workers, could provide a more complete view of the challenges and solutions in psychosocial support within the educational system. Studies that associate the academic trajectory of youth in vulnerable situations with their transition to adulthood and the acquisition of autonomy are essential. Studies focused on specific situations of vulnerability can favor more targeted and in-depth analyses. The direct participation of young people should be a priority when designing the research study. They should be the protagonists, they should have a voice, and it is necessary to ensure that this voice is truly heard, respected, valued, and honestly and carefully treated and shared.

## Figures and Tables

**Table 1:** Participants in the interviews.

ID	Gender	Age	Birthplace	Occupation	Completed Education Level
E1	Male	25	Other countries	Studying and working	University Degree
E2	Female	20	Spain	Studying	CSE
E3	Male	18	Spain	Studying	High school
E4	Female	24	Other countries	Studying	University Degree
E5	Female	26	Spain	Working	University Degree
E6	Male	24	Other countries	Studying	CSE
E7	Male	21	Other countries	Studying and working	CSE
E8	Male	18	Other countries	Studying and working	High school
E9	Female	24	Other countries	Studying and working	VT
E10	Female	23	Other countries	Studying and working	High school
E11	Male	18	Spain	Studying	High school
E12	Female	17	Spain	Studying	CSE

Note: VT = vocational training; CSE = compulsory secondary education.

**Table 2:** Psychosocial factors that influence young people’s formal education trajectories.

Code	F.	Subcodes	F.
Caregivers’ valuation of formal education (coverage: 11)	29	Comprehensive and supportive attitude (coverage: 10)	24
		Low support and valuation (coverage: 4)	5
Respondents’ valuation of formal education (coverage: 6)	6	Important for the future (coverage: 4)	4
		Important to get a good job (coverage: 2)	2
Positive school experiences (coverage: 10)	42	About success in studies (coverage: 8)	17
		Positive relationships w/classmates (coverage: 3)	4
		Positive relationships w/teachers or school professionals (coverage: 7)	21
Negative school experiences (coverage: 9)	31	About failures in studies (coverage: 7)	11
		Negative relationships w/classmates (coverage: 5)	9
		Negative relationships w/teachers or school professionals (coverage: 5)	11
Personal characteristics that lead to success (coverage: 12)	28	Positive attitudes to problems (coverage: 7)	10
		Individual protective factors (coverage: 10)	18
Support networks (coverage: 11)	33	Family or friends (coverage: 10)	17
		Institutions or professionals (coverage: 8)	16
Formal education obstacles (coverage: 10)	23	Educational system (coverage: 2)	6
		Language (coverage: 4)	5
		Legal issues (coverage: 2)	2
		Provenance (coverage: 2)	2
		Vulnerability situation (coverage: 4)	8
Suggestions to favor psychosocial support in formal education contexts (coverage: 4)	15	Educational system structure (coverage: 4)	10
		Teachers’ competencies or approach (coverage: 3)	5
Plans for the future (coverage: 12)	20	Doubts or inaccurate definition (coverage: 7)	10
		Formation goals (coverage: 4)	4
		Job goals (coverage: 2)	4
		Relationship goals (coverage: 3)	2

## Data Availability

The data are not publicly available due to privacy or ethical restrictions.
